# Place of death among individuals with chronic respiratory diseases in China: Trends and associated factors between 2014 and 2020

**DOI:** 10.3389/fpubh.2023.1043534

**Published:** 2023-02-20

**Authors:** Xunliang Tong, Wei Wang, Xinyue Zhang, Peng Yin, Enying Gong, Yanming Li, Maigeng Zhou

**Affiliations:** ^1^Department of Pulmonary and Critical Care Medicine, National Center of Gerontology, Beijing Hospital, Institute of Geriatric Medicine, Chinese Academy of Medical Sciences, Beijing, China; ^2^National Center for Chronic and Non-Communicable Disease Control and Prevention, Chinese Center for Disease Control and Prevention, Beijing, China; ^3^Peking Union Medical College Graduate School, Chinese Academy of Medical Sciences and Peking Union Medical College, Beijing, China; ^4^School of Population Medicine and Public Health, Chinese Academy of Medical Sciences and Peking Union Medical College, Beijing, China

**Keywords:** asthma, chronic obstructive pulmonary disease (COPD), epidemiology, place of death, trends and associated factors

## Abstract

**Background:**

Chronic respiratory disease (CRD) is a common cause of mortality in China, but little is known about the place of death (POD) among individuals with CRD.

**Methods:**

Information about CRD-caused deaths was obtained from the National Mortality Surveillance System (NMSS) in China, covering 605 surveillance points in 31 provinces, autonomous regions, and municipalities. Both individual- and provincial-level characteristics were measured. Multilevel logistic regression models were built to evaluate correlates of hospital CRD deaths.

**Results:**

From 2014 to 2020, a total of 1,109,895 individuals who died of CRD were collected by the NMSS in China, among which home was the most common POD (82.84%), followed by medical and healthcare institutions (14.94%), nursing homes (0.72%), the way to hospitals (0.90%), and unknown places (0.59%). Being male, unmarried, having a higher level of educational attainment, and being retired personnel were associated with increased odds of hospital death. Distribution of POD differed across the provinces and municipalities with different development levels, also presenting differences between urban and rural. Demographics and individual socioeconomic status (SES) explained a proportion of 23.94% of spatial variations at the provincial level. Home deaths are the most common POD (>80%) among patients with COPD and asthma, which are the two major contributors to CRD deaths.

**Conclusion:**

Home was the leading POD among patients with CRD in China in the study period; therefore, more attention should be emphasized to the allocation of health resources and end-of-life care in the home setting to meet the increasing needs among people with CRD.

## Introduction

Non-communicable diseases (NCDs) are the leading cause of death and the main contributors to premature deaths worldwide (1, 2). As a crucial category of NCDs, chronic respiratory diseases (CRD) have been the third leading cause of death globally, ranked behind cardiovascular diseases and cancers, but receive less attention than other NCDs (3). According to the Global Burden of Disease estimation, CRD caused 3.97 million deaths globally in 2019, among which chronic obstructive pulmonary disease (COPD) and asthma are the two main causes of CRD deaths (4). The World Health Organization estimates that COPD caused about 3.23 million deaths in 2019, responsible for ~6% of total deaths, of which more than 90% occurred in low-income and middle-income countries (5). Asthma affected an estimated 262 million people in 2019 and caused 461,000 deaths, responsible for 11.6% of CRD deaths and 1.1% of NCD deaths (4). In China, CRD has always been one of the main causes of death, accounting for 10.62% of total deaths according to the monitoring from the death surveillance points system of the Chinese Center for Disease Control and Prevention (CDC) in 2019 (6). It was estimated that there were 1.085 million CRD deaths in China in 2019, accounting for 27.3% of global CRD deaths (7). Moreover, the challenge brought by China's growing population to CRD is undeniable.

Place of death (POD) is one of the important indicators for the quality of end-of-life (EOL) care at the population level. Analyzing the POD of CRD could inform the major needs of patients with CRD and provide key information for health resource allocations. For example, a previous study conducted in England found that the hospital was the most common POD for people with advanced respiratory diseases, and the end-of-life care strategy has contributed to a tangible reduction in hospital-based deaths between 2001 and 2014 (8). Similarly, a trend analysis study conducted in the US found that home deaths were rising among decedents from chronic lung diseases between 2003 and 2017, which indicated an increasing need for quality EOL care in the US (9). Previous studies also illustrate some factors that were associated with the POD, including sociodemographic characteristics, patients' preferences, and cost (10, 11). A retrospective review of COPD deaths in Spain also found sex differences in POD with a higher proportion of deaths occurring at home among women (12).

However, few studies analyzed the trends of POD of CRD deaths in low- and middle-income countries. In China, there are only a few studies that described the burden and death rates of CRD (7); while to the best of our knowledge, the POD of CRD has not been well-studied in China. Without evidence on the characteristics and trends of POD of CRD deaths, relevant policies are hard to be made on whether the allocation of healthcare resources could sufficiently fulfill the needs for EOL care. To address these gaps, our study aims to assess POD trends between 2014 and 2020 and describe factors associated with POD among CRD decedents in China.

## Study design and methods

### Data sources

Data for CRD deaths were extracted from the National Mortality Surveillance System (NMSS) managed by the Chinese CDC.

The NMSS combines the disease surveillance point system and the vital registration system and monitors the mortality cases from 605 surveillance points in all 31 provinces, autonomous regions, and municipalities in mainland China, covering over 300 million individuals in China which accounts for 24% of the population (13). The NMSS routinely collects individual details of death information in real-time through an internet-based approach. Detailed descriptions of NMSS representativeness, determination, surveillance points selection, coding and determining the underlying cause of deaths, and quality control have been reported elsewhere (14).

### Data quality control

The general quality of data from the NMSS could be found in the previous study (13), which also demonstrated the higher quality of the data since 2013. We have carefully designed rules to ensure data quality with the following key approaches. The quality of data from each of the surveillance points and at the national level has been reviewed, compared, and evaluated comprehensively. Following the previous study (14), we set the exclusion criteria by excluding the surveillance points if the crude all-cause mortality rate is lower than 5%. In addition, we also checked the details of the death cases and excluded the cases where key variables, including age, sex, and POD, were missing (Supplementary Table 1).

### Data extraction on CRD-related deaths

Underlying COD in the NMSS was recorded by following the International Classification of Disease 10th Edition (ICD-10). We classified total CRD as COPD, asthma, pneumoconiosis, interstitial lung disease, and other CRDs. All deaths between 2014 and 2020 in which CRD was identified as underlying COD were extracted for this study.

### POD and co-variates

The place of death was recorded in the death certificate with the options of hospitals, emergency departments, homes, nursing homes, on the way to hospitals, other places, and unknown. We classified these categories into five groups: hospitals (including emergency departments), homes, nursing homes, on the way to hospitals, and others/unknown. For explanatory variables, we included residency (in which we defined districts as urban areas and counties as rural areas), demographics (sex, age, ethnicity, and marital status), socioeconomic status (SES, including the level of educational attainment and occupation), and disease-related factors (underlying cause of death and highest diagnostic institutions). We analyzed age (years old) as an ordered four category (0–14, 15–64, 65–84, and 85 and above) rather than a continuous variable to facilitate interpretation and comparison with other studies (15–17). Besides, socioeconomic contextual factors at the provincial level were measured and classified, including region (western, central, and eastern), per capita gross domestic product (GDP) (in quartiles of GDP, 10,000 RMB per person, about US $1,430 per person), average years of education attainment (in quartiles of years), and number of beds in healthcare institutions (in quartiles of units per 100,000 persons). To analyze associated factors with POD, we also included several socioeconomic development and equity metrics in the subgroup analysis: urbanization rate (%), the unemployment rate in urban areas (%), Engel's coefficient, Gini index, number of medical technical personnel in healthcare institutions (units per 100,000 persons), and sex ratio and gross dependency ratio (%). For GDP at the provincial level, in the consideration of discount rate during the study period, we used a 3% (0–6% for recommended range) discount rate to translate cash (GDP) at the end of each time point (from 2014 to 2020) into present value in 2014 (18). The Engel's coefficient, which equals to the proportion that points to the food expenditure occupied total consumption expenditure in dweller family, and the GINI index, which refers to the proportion of the income used for an unequal distribution, were extracted from the national and provincial statistics yearbook for each province and the values were classified into quartiles in the analysis.

### Statistical analysis

We first presented POD trends of CRD deaths by important characteristics during 2014–2020. We used chi-square tests to compare the differences in characteristics among nominal categories and logistic regression to test their trends for ordered variables. We performed a poison model, which was adjusted for age at death, sex, and provincial area, to examine the changes in the percentage of POD among CRD deaths and its subcategories at the national level during 2014–2020.

Subsequently, we conducted multilevel logistic regression to explore the individual factors and socioeconomic contextual correlates of hospital CRD deaths, thus we defined home, nursing homes, on the way to hospitals, and others/unknown as “out-of-hospital CRD deaths.” Several models were constructed separately. First, a two-level null model (Model 1) that included fixed and random intercepts were fitted to investigate spatial variations across multiple scales, among which, random intercepts accounted for the clustering of participants (level 1) within provinces (level 2). Random effects at the individual level thus were translated into median odds ratio (MOR) which indicated the median value of the odds between the area with the highest outcome probability and the area with the lowest (19). To explore to what extent those personal and contextual factors explained the spatial variations of hospital CRD deaths, demographics (Model 2), SES (Model 3), underlying COD (Model 4), and contextual phenomena (Model 5) were stepwise entered into the multilevel logistic model. The proportional change in variance was calculated for each step, which reflects the proportion of variation that can be explained by newly added variables to the model (20, 21). Afterward, we added two-way interaction terms of the individual-level factors and separate provincial-level characteristics to the models to examine whether the effects of contextual variables on hospital CRD deaths were modified by personal variables (Model 6) (22, 23). For detailed CRD subcategories, we performed a similar analysis among COPD and asthma deaths, respectively, to identify associated factors in the two leading causes of CRD deaths.

A *P* < 0.05 was considered statistically significant and all tests were two-sided. The analysis was performed in SAS 9.4 (SAS Institute Inc., Cary, North Carolina, USA).

## Results

### POD distribution and trends among CRD deaths

During 2014–2020, a total of 1,109,895 individuals who died of CRD were collected by NMSS in China, ranging from 178,641 in 2014 to 134,380 in 2020, as shown in Table 1. Among these CRD deaths, 1,057,454 (95.28%) deaths were caused by COPD, while 30,030 (2.71%) deaths were caused by asthma, and the rest was caused by pneumoconiosis (0.76%), ILD (0.98%), and other chronic pulmonary diseases (0.28%). For all CRD-caused deaths accounted during the study period, the home was the leading POD (82.84%), followed by medical and healthcare institutions (14.94%), on the way to hospitals (0.90%), or at unknown places (0.59%).

**Table 1:** Characteristics of POD distribution among CRD deaths from the NMSS in China, 2014–2020 [Death counts (person), %].

**Characteristics**	**Total**	**Medical and healthcare institutions**	**Out of hospital**			
			**Home**	**Nursing homes**	**On the way to hospitals**	**Others/unknown**
Total	1,109,895 (100.00)	165,861 (14.94)	919,438 (82.84)	8,022 (0.72)	10,017 (0.90)	6,557 (0.59)
**Location**						
Rural	809,111 (72.90)	86,901 (52.39)	706,812 (76.87)	5,654 (70.48)	5,416 (54.07)	4,328 (66.01)
Urban	300,784 (27.10)	78,960 (47.61)	212,626 (23.13)	2,368 (29.52)	4,601 (45.93)	2,229 (33.99)
*P*_fordifference_	<0.001	<0.001	<0.001	<0.001	<0.001	<0.001
**Region^a^**						
Western	429,724 (38.72)	54,538 (32.88)	365,794 (39.78)	2,925 (36.46)	3,056 (30.51)	3,411 (52.02)
Central	328,722 (29.62)	60,588 (36.53)	261,044 (28.39)	2,904 (36.20)	2,494 (24.90)	1,692 (25.80)
Eastern	351,449 (31.67)	50,735 (30.59)	292,600 (31.82)	2,193 (27.34)	4,467 (44.59)	1,454 (22.17)
*P*_fordifference_	<0.001	<0.001	<0.001	<0.001	<0.001	<0.001
**Sex**						
Male	649,771 (58.54)	108,125 (65.19)	525,445 (57.15)	5,070 (63.20)	6,893 (68.81)	4,238 (64.63)
Female	460,124 (41.46)	57,736 (34.81)	393,993 (42.85)	2,952 (36.80)	3,124 (31.19)	2,319 (35.37)
*P*_fordifference_	<0.001	<0.001	<0.001	<0.001	<0.001	<0.001
**Age groups, years old**						
0–14	244 (0.02)	87 (0.05)	119 (0.01)	28 (0.35)	0 (0)	10 (0.15)
15–64	97,814 (8.81)	19,488 (11.75)	74,812 (8.14)	1,410 (17.58)	645 (6.44)	1,459 (22.25)
65–84	668,302 (60.21)	99,133 (59.77)	554,972 (60.36)	4,824 (60.13)	5,637 (56.27)	3,736 (56.98)
85 and above	343,535 (30.95)	47,153 (28.43)	289,535 (31.49)	1,760 (21.94)	3,735 (37.29)	1,352 (20.62)
*P*_fortrend_	<0.001	<0.001	<0.001	<0.001	<0.001	<0.001
**Ethnicity**						
Han	1,033,440 (93.11)	160,581 (96.82)	849,534 (92.40)	7,474 (93.17)	9,707 (96.91)	6,144 (93.70)
Other ethnics	76,455 (6.89)	5,280 (3.18)	69,904 (7.60)	548 (6.83)	310 (3.09)	413 (6.30)
*P*_fordifference_	<0.001	<0.001	<0.001	<0.001	<0.001	<0.001
**Marital status**						
Married	651,104 (58.66)	115,085 (69.39)	524,126 (57.01)	5,466 (68.14)	2,531 (25.27)	3,896 (59.42)
Unmarried	30,498 (2.75)	5,165 (3.11)	21,762 (2.37)	244 (3.04)	2,882 (28.77)	445 (6.79)
Widowed/divorced	422,071 (38.03)	44,411 (26.78)	369,119 (40.15)	2,238 (27.90)	4,497 (44.89)	1,806 (27.54)
Unknown	6,222 (0.56)	1,200 (0.72)	4,431 (0.48)	74 (0.92)	107 (1.07)	410 (6.25)
*P*_fordifference_	<0.001	<0.001	<0.001	<0.001	<0.001	<0.001
**Education**						
Junior high school and below	1,062,221 (95.70)	140,784 (84.88)	898,794 (97.75)	7,277 (90.71)	9,268 (92.52)	6,098 (93.00)
Senior high school	36,816 (3.32)	17,600 (10.61)	17,723 (1.93)	592 (7.38)	534 (5.33)	367 (5.60)
College and above	10,858 (0.98)	7,477 (4.51)	2,921 (0.32)	153 (1.91)	215 (2.15)	92 (1.40)
*P*_fortrend_	<0.001	<0.001	<0.001	<0.001	<0.001	<0.001
**Occupation**						
Agricultural-related personnel	906,869 (81.71)	74,700 (45.04)	816,686 (88.82)	5,556 (69.26)	5,571 (55.62)	4,356 (66.43)
Retired	92,716 (8.35)	49,680 (29.95)	39,637 (4.31)	995 (12.40)	1,870 (18.67)	534 (8.14)
Unemployment/student	36,785 (3.31)	11,279 (6.80)	23,911 (2.60)	428 (5.34)	856 (8.55)	311 (4.74)
Worker/self-employed/enterprise manager	22,263 (2.01)	9,018 (5.44)	12,114 (1.32)	387 (4.82)	471 (4.70)	273 (4.16)
Professional/staff/civil servant	7,726 (0.70)	4,338 (2.62)	3,034 (0.33)	122 (1.52)	143 (1.43)	89 (1.36)
Others/unknown	43,536 (3.92)	16,846 (10.16)	24,056 (2.62)	534 (6.66)	1,106 (11.04)	994 (15.16)
*P*_fordifference_	<0.001	<0.001	<0.001	<0.001	<0.001	<0.001
**Underlying cause of death**						
Chronic obstructive pulmonary disease	1,057,454 (95.28)	152,041 (91.67)	882,745 (96.01)	7,256 (90.45)	9,494 (94.78)	5,918 (90.25)
Asthma	30,030 (2.71)	4,120 (2.48)	24,620 (2.68)	487 (6.07)	402 (4.01)	401 (6.12)
Pneumoconiosis	8,399 (0.76)	2,100 (1.27)	6,104 (0.66)	92 (1.15)	44 (0.44)	59 (0.90)
Interstitial lung disease	10,925 (0.98)	6,947 (4.19)	3,732 (0.41)	118 (1.47)	58 (0.58)	70 (1.07)
Other chronic respiratory diseases	3,087 (0.28)	653 (0.39)	2,237 (0.24)	69 (0.86)	19 (0.19)	109 (1.66)
*P*_fordifference_	<0.001	<0.001	<0.001	<0.001	<0.001	<0.001
**Highest diagnostic institutions**						
Village clinics	35,964 (3.24)	306 (0.18)	35,199 (3.83)	121 (1.51)	189 (1.89)	149 (2.27)
Primary institutions	186,038 (16.76)	12,533 (7.56)	169,493 (18.43)	1,119 (13.95)	2,034 (20.31)	859 (13.10)
Secondary institutions	560,215 (50.47)	70,415 (42.45)	478,856 (52.08)	4,063 (50.65)	3,898 (38.91)	2,983 (45.49)
Tertiary institutions	295,703 (26.64)	81,586 (49.19)	206,387 (22.45)	2,550 (31.79)	3,383 (33.77)	1,797 (27.41)
Other institutions	5,613 (0.51)	943 (0.57)	4,018 (0.44)	41 (0.51)	240 (2.40)	371 (5.66)
Undiagnosed	26,362 (2.38)	78 (0.05)	25,485 (2.77)	128 (1.60)	273 (2.73)	398 (6.07)
*P*_fordifference_	<0.001	<0.001	<0.001	<0.001	<0.001	<0.001

a Region: Western: Inner Mongolia, Guangxi, Chongqing, Sichuan, Guizhou, Yunnan, Tibet, Shaanxi, Gansu, Qinghai, Ningxia, Xinjiang; Central: Shanxi, Jilin, Heilongjiang, Anhui, Jiangxi, Henan, Hubei, and Hunan; Eastern: Beijing, Tianjin, Hebei, Liaoning, Shanghai, Jiangsu, Zhejiang, Fujian, Shandong, Guangdong, and Hainan.

**Figure 1:**
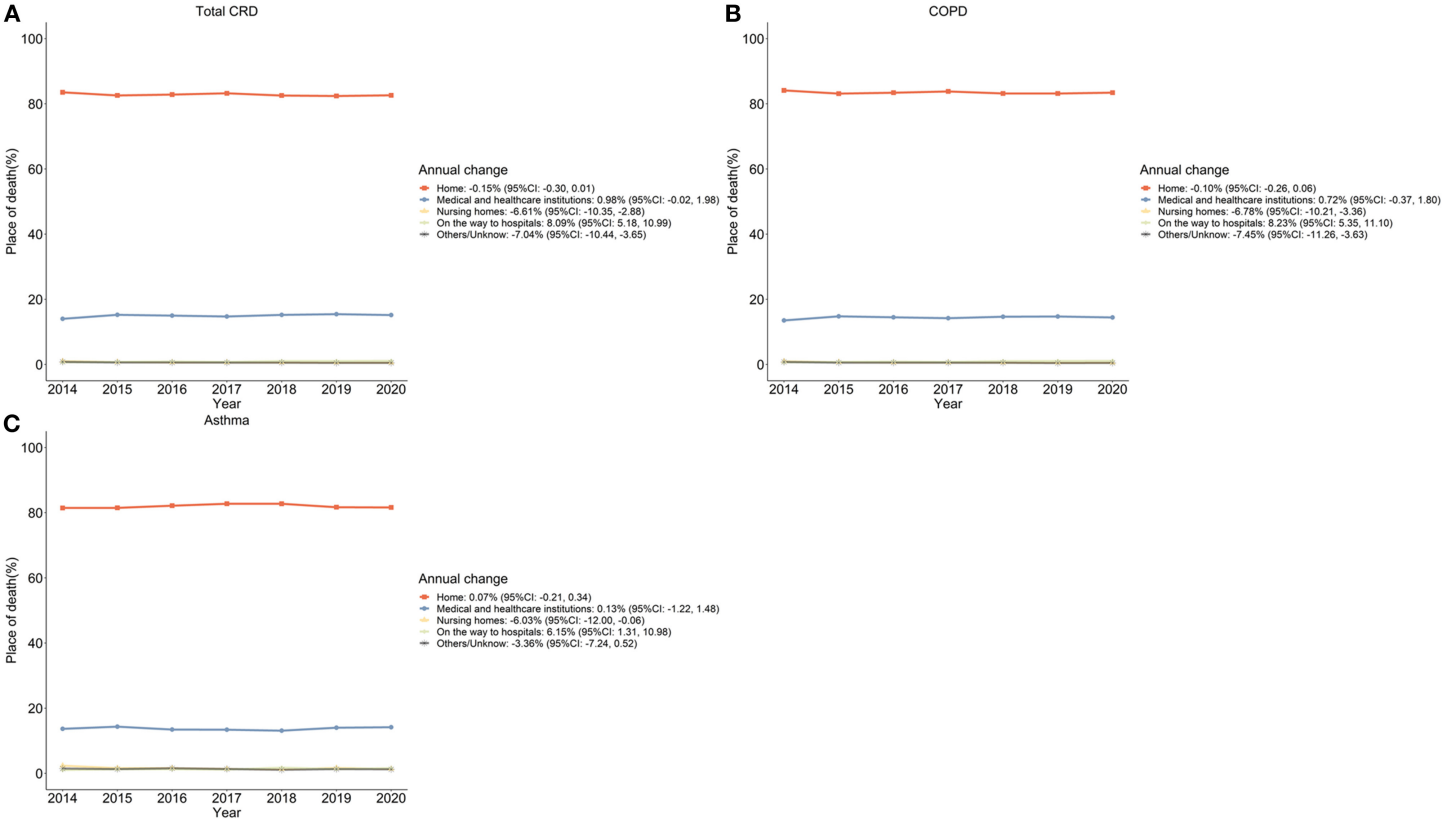
The proportion of subcategories of POD among CRD deaths in China, 2014–2020 (%). **(A)** Total CRD-caused deaths; **(B)** COPD-caused deaths; **(C)** Asthma-caused deaths.

The changes in POD among CRD deaths presented a relatively stable tendency during the observation period (Figure 1). For specific CRDs, homes and hospitals are the top two PODs. During 2014–2020, COPD and asthma shared a pattern of increasing hospital and on-the-way-to-hospital deaths as well as decreasing nursing homes and other/unknown deaths. However, a slight decrease in home deaths occurred in COPD deaths (Annual change −0.10%) while an increase was observed in deaths due to asthma (Annual change 0.07%) (Supplementary Table 2).

### The individual and contextual factors associated with the POD of CRD deaths

**Figure 2:**
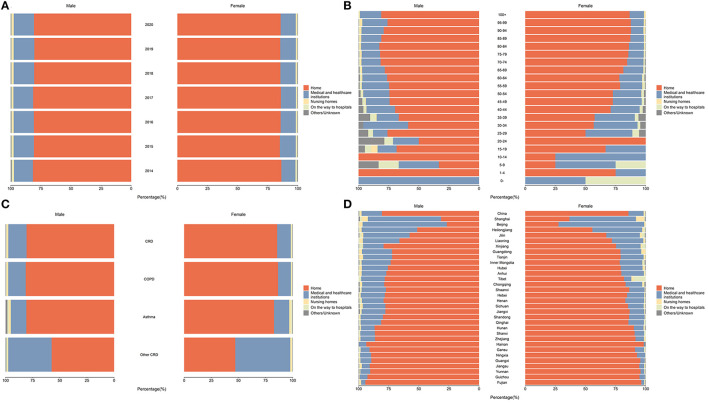
The distribution of POD among CRD deaths in China, by sex (%). **(A)** Distribution of POD among CRD deaths in China, 2014–2020; **(B)** Distribution of POD among CRD deaths in China, by age, 2020; **(C)** Distribution of POD among CRD deaths in China, by CRD subcategories, 2020; **(D)** Distribution of POD among CRD deaths in China, by province, 2020.

The disparities of POD distribution by important sociodemographic characteristics were also presented in Table 1, and all of them showed significant differences between each group. As shown in Figure 2, the POD trends were similar between women and men by year of death and age groups, but in general, a higher proportion of women died at home and men died more often in hospitals. The proportion of home deaths raised with age as an adult and elderly patients with CRD were found to be more likely to die at home; while major POD for infant and adolescent patients were in hospitals. In addition, we observed higher proportions of hospital deaths among unmarried people and people with college and above education.

**Figure 3:**
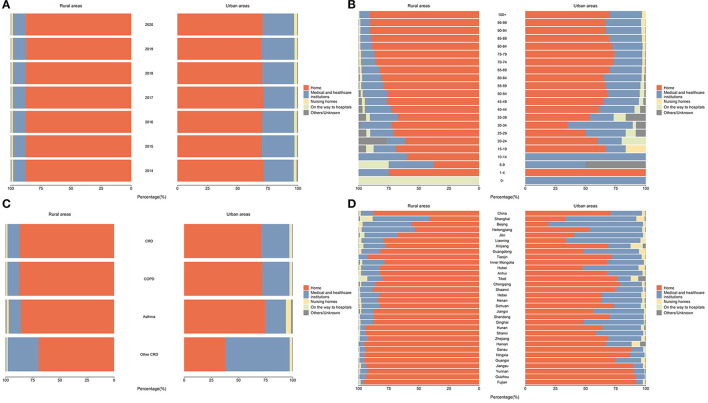
The distribution of POD among CRD deaths in China, by rurality status (%). **(A)** Distribution of POD among CRD deaths in China, 2014–2020; **(B)** Distribution of POD among CRD deaths in China, by age, 2020; **(C)** Distribution of POD among CRD deaths in China, by CRD subcategories, 2020; **(D)** Distribution of POD among CRD deaths in China, by province, 2020.

We also found different POD distributions of CRD deaths by their residency, in terms of rural or urban settings and across provinces and municipalities (Figure 3). In 2020, patients with CRD residing in rural areas were more likely to die at home, while patients residing in urban areas were more likely to die in a hospital and the percentage of dying in nursing homes, on the way to hospitals, and others/unknown also showed a slightly higher level. Across provinces, Beijing (70.39%) and Shanghai (58.49%) were the top two municipalities with the highest proportion of hospital deaths, while Hainan (95.51%) and Fujian (95.16%) were the top two provinces with the highest proportion of home deaths. The proportion of deaths on the way to hospitals and others/unknown varies little between provinces.

### Multilevel analysis on factors associated with POD in CRD

Multilevel modeling results demonstrated the associated factors considering both individual-level factors and contextual factors (Table 2). The differences in demographics (age, sex, ethnicity, and marital status) and individual SES (education and occupation) substantially contributed to explaining 23.94% of spatial variations among hospital CRD deaths below the province scale when compared to Model 1 with Model 3. Meanwhile, the result of MOR of Model 3 (2.36 >1) also indicated significant regional variations and helped to explain individual differences in POD. In Model 4, 23.94% of the variation (the MOR was 2.17) were explained by both individual factors and underlying cause of death. In Model 5, 21.53% of the variation (the MOR was 2.20) was explained by both individual factors and contextual factors such as regions, GDP, average years of educational attainment, and number of beds in healthcare institutions locally. Specifically, patients with CRD who were female (OR: 1.01, 95% CI: 0.99–1.02), unmarried (OR: 1.21, 95% CI: 1.17–1.25), lived in urban areas (OR: 1.46, 95% CI: 1.44–1.48), with college and above educational background (OR: 2.44, 95% CI: 2.33–2.55), the retired personnel (OR: 9.53, 95% CI:9.34–9.69), lived in the central areas of the country (OR: 1.32, 95% CI: 0.61–2.87) have a higher probability of dying in hospitals compared with their counterparts. Besides, the probability of CRD deaths in hospitals was lower among those who were widowed/divorced (OR: 0.59, 95% CI: 0.58–0.60), ethnic minorities (OR: 0.49, 95% CI: 0.47–0.51), and died of COPD or asthma.

**Table 2:** Associated factors of hospital CRD deaths from the NMSS in China, 2014–2020: estimated from multilevel logistics regression.

**Factors**	**Model 1**	**Model 2**	**Model 3**	**Model 4**	**Model 5**	**Model 6**
	**OR (95% CI)**	**OR (95% CI)**	**OR (95% CI)**	**OR (95% CI)**	**OR (95% CI)**	**OR (95% CI)**
**Fixed effect**						
Constant	0.18(0.13 ~ 0.24)	0.20(0.14 ~ 0.27)	0.14(0.11 ~ 0.20)	0.29(0.21 ~ 0.39)	0.30(0.19 ~ 0.48)	0.30(0.19 ~ 0.49)
Year (2014–2020)	–	1.01(1.01 ~ 1.01)^*^	1.00(1.00 ~ 1.00)^*^	1.00(1.00 ~ 1.00)^*^	1.01(1.00 ~ 1.02)^*^	1.01(1.00 ~ 1.01)^*^
**Location (reference: rural)**						
Urban	–	2.99(2.95 ~ 3.03)^*^	1.46(1.43 ~ 1.48)^*^	1.46(1.44 ~ 1.48)^*^	1.46(1.44 ~ 1.48)^*^	1.46(1.45 ~ 1.48)^*^
**Sex (reference: male)**						
Female	–	0.79(0.79 ~ 0.80)^*^	1.01(0.99 ~ 1.02)	1.01(1.00 ~ 1.03)^*^	1.02(1.00 ~ 1.03)^*^	1.02(1.01 ~ 1.03)^*^
**Age groups, years old (reference: 0–14)**						
15–64	–	0.32(0.24 ~ 0.42)^*^	1.08(0.82 ~ 1.44)	1.34(1.01 ~ 1.79)^*^	1.34(1.01 ~ 1.79)^*^	1.34(1.01 ~ 1.79)^*^
65–84	–	0.25(0.19 ~ 0.33)^*^	0.76(0.58 ~ 1.01)	0.96(0.72 ~ 1.29)	0.97(0.72 ~ 1.29)	0.97(0.72 ~ 1.29)
85 and above	–	0.26(0.20 ~ 0.35)^*^	0.71(0.54 ~ 0.94)^*^	0.90(0.68 ~ 1.20)	0.90(0.68 ~ 1.20)	0.90(0.68 ~ 1.20)
**Ethnicity (reference: han)**						
Other ethnics	–	0.45(0.43 ~ 0.47)^*^	0.49(0.47 ~ 0.51)^*^	0.49(0.47 ~ 0.50)^*^	0.49(0.47 ~ 0.51)^*^	0.49(0.47 ~ 0.50)^*^
**Marital status (reference: married)**						
Unmarried	–	0.91(0.88 ~ 0.94)^*^	1.19(1.15 ~ 1.23)^*^	1.21(1.17 ~ 1.25)^*^	1.21(1.17 ~ 1.25)^*^	1.21(1.17 ~ 1.25)^*^
Widowed/divorced	–	0.55(0.55 ~ 0.56)^*^	0.59(0.58 ~ 0.59)^*^	0.59(0.58 ~ 0.60)^*^	0.59(0.58 ~ 0.60)^*^	0.59(0.58 ~ 0.60)^*^
Unknown	–	0.93(0.87 ~ 1.00)^*^	0.85(0.79 ~ 0.91)^*^	0.85(0.80 ~ 0.92)^*^	0.85(0.79 ~ 0.92)^*^	0.85(0.79 ~ 0.91)^*^
**Education (reference: junior high school and below)**						
Senior high school	–	–	1.61(1.57 ~ 1.65)^*^	1.61(1.57 ~ 1.65)^*^	1.61(1.56 ~ 1.65)^*^	1.61(1.56 ~ 1.66)^*^
College and above	–	–	2.47(2.36 ~ 2.59)^*^	2.44(2.33 ~ 2.55)^*^	2.44(2.33 ~ 2.55)^*^	2.44(2.33 ~ 2.55)^*^
**Occupation (reference: agricultural-related personnel)**						
Retired	–	–	9.74(9.56 ~ 9.92)^*^	9.51(9.33 ~ 9.69)^*^	9.52(9.35 ~ 9.70)^*^	9.53(9.34 ~ 9.69)^*^
Unemployment/student	–	–	4.14(4.03 ~ 4.24)^*^	4.14(4.03 ~ 4.25)^*^	4.14(4.03 ~ 4.25)^*^	4.12(4.03 ~ 4.25)^*^
Worker/self-employed/enterprise manager	–	–	5.17(5.01 ~ 5.33)^*^	5.00(4.84 ~ 5.16)^*^	5.00(4.84 ~ 5.16)^*^	4.99(4.83 ~ 5.16)^*^
Professional/staff/civil servant	–	–	7.93(7.53 ~ 8.36)^*^	7.76(7.36 ~ 8.17)^*^	7.75(7.35 ~ 8.17)^*^	7.75(7.35 ~ 8.17)^*^
Others/unknown	–	–	4.82(4.71 ~ 4.94)^*^	4.79(4.68 ~ 4.91)^*^	4.82(4.72 ~ 4.94)^*^	4.82(4.71 ~ 4.93)^*^
**Underlying cause of death (reference: other CRD)**						
COPD	–	–	–	0.52(0.50 ~ 0.54)^*^	0.52(0.51 ~ 0.54)^*^	0.52(0.51 ~ 0.55)^*^
Asthma	–	–	–	0.34(0.31 ~ 0.35)^*^	0.34(0.32 ~ 0.36)^*^	0.34(0.32 ~ 0.35)^*^
**Region (reference: western)** ^a^						
Central	–	–	–	–	1.32(0.60 ~ 2.86)	1.32(0.61 ~ 2.87)
Eastern	–	–	–	–	0.74(0.36 ~ 1.52)	0.74(0.36 ~ 1.51)
**GDP, 10,000 RMB per person (reference: Q1**,<**2.55)**						
Q2 (2.55–4.51)	–	–	–	–	1.18(1.09 ~ 1.28)^*^	1.18(1.08 ~ 1.28)^*^
Q3 (4.52–6.06)	–	–	–	–	1.24(1.13 ~ 1.35)^*^	1.24(1.13 ~ 1.36)^*^
Q4 (≥6.07)	–	–	–	–	1.28(1.16 ~ 1.42)^*^	1.28(1.16 ~ 1.41)^*^
**Average years of education attainment, years (reference: Q1**,<**8.83)**						
Q2 (8.84–9.27)	–	–	–	–	0.88(0.86 ~ 0.91)^*^	0.88(0.85 ~ 0.91)^*^
Q3 (9.28–9.67)	–	–	–	–	0.99(0.93 ~ 1.03)	0.99(0.94 ~ 1.03)
Q4 (≥9.68)	–	–	–	–	0.99(0.94 ~ 1.06)	0.99(0.94 ~ 1.04)
**Number of beds in healthcare institutions, units per 100,000 persons (references: Q1**,<**4.93)**						
Q2 (4.94–5.60)	–	–	–	–	0.87(0.85 ~ 0.89)^*^	0.87(0.84 ~ 0.89)^*^
Q3 (5.61–6.37)	–	–	–	–	0.83(0.80 ~ 0.87)^*^	0.84(0.80 ~ 0.88)^*^
Q4 (≥6.38)	–	–	–	–	0.82(0.78 ~ 0.86)^*^	0.81(0.76 ~ 0.85)^*^
**GDP** × **junior high school and below (reference)**						
GDP × senior high school	–	–	–	–	–	1.08(1.02–1.15)^*^
GDP × college and above	–	–	–	–	–	1.12(1.05–1.21)^*^
**GDP** × **agricultural-related personnel (reference)**						
GDP × retired	–	–	–	–	–	1.07(1.02–1.01)^*^
GDP × unemployment/student	–	–	–	–	–	1.02(0.98–1.07)^*^
GDP × worker/self–employed/enterprise manager	–	–	–	–	–	1.09(1.03–1.04)^*^
GDP × professional/staff/civil servant	–	–	–	–	–	1.21(1.13–1.30)^*^
GDP × others/unknown	–	–	–	–	–	1.05(1.01–1.09)^*^
**Random effects**						
Variance among provinces (SE)	0.88 (0.23)	0.81 (0.21)	0.67 (0.17)	0.67 (0.17)	0.69 (0.19)	0.68 (0.19)
MOR	2.44	2.36	2.18	2.17	2.20	2.20
PCV (%)	–	7.83	23.94	24.31	21.53	22.80

OR, odds ratio; CI, confidence interval; Q1, 1st quantile; Q2, 2nd quantile; Q3, 3rd quantile; Q4, 4th quantile; SE, standard error; MOR, median odds ratio; PCV, Proportional change in variance.

* P < 0.05.

a Region: Western: Inner Mongolia, Guangxi, Chongqing, Sichuan, Guizhou, Yunnan, Tibet, Shaanxi, Gansu, Qinghai, Ningxia, Xinjiang; Central: Shanxi, Jilin, Heilongjiang, Anhui, Jiangxi, Henan, Hubei, and Hunan; Eastern: Beijing, Tianjin, Hebei, Liaoning, Shanghai, Jiangsu, Zhejiang, Fujian, Shandong, Guangdong, and Hainan.

In Model 6, a comparatively higher level of GDP was estimated to still be positively associated with hospital CRD deaths, and this relationship was increased by adding up individual SES (GDP at its Q2 increased from OR: 1.81, 95% CI:1.08–1.28 to OR: 1.28, 95% CI:1.16–1.41). Besides, all models also examined nearly unchanged odds (OR: 0.99, 95% CI: 0.98–1.00) of hospital CRD deaths nationally over time, and NMSS expansion in 2014 increased the probability of reporting hospital deaths (OR: 1.01, 95% CI: 1.00–1.01).

### POD distribution in categories of CRD

**Figure 4:**
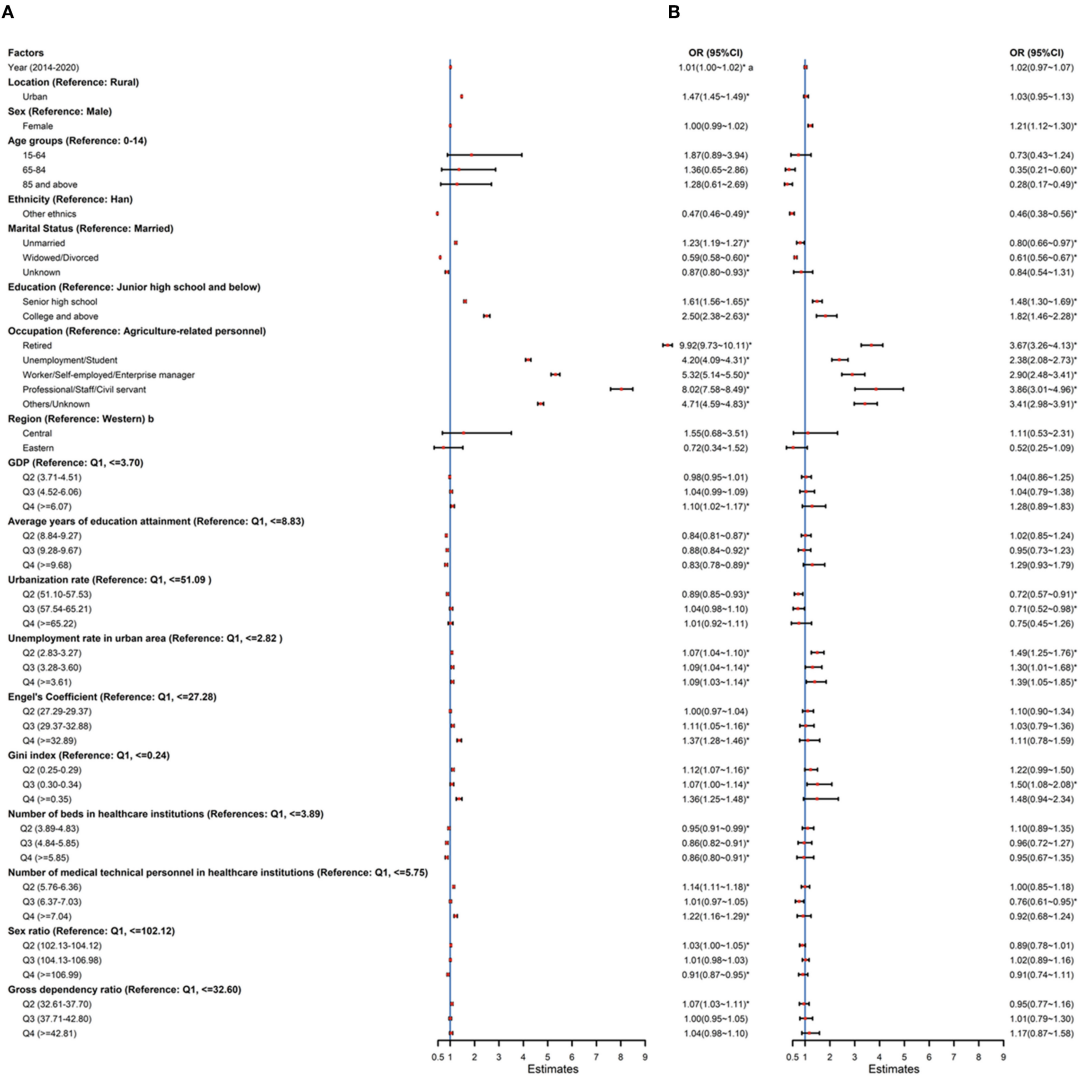
Associated factors of hospital COPD and asthma deaths in China, 2014–2020: estimated from multilevel logistics regression. OR, odds ratio; CI, confidence interval; Q1, 1st quantile; Q2, 2nd quantile; Q3, 3rd quantile; Q4, 4th quantile. ^a^^*^P < 0.05. ^b^Region: Western: Inner Mongolia, Guangxi, Chongqing, Sichuan, Guizhou, Yunnan, Tibet, Shaanxi, Gansu, Qinghai, Ningxia, Xinjiang; Central: Shanxi, Jilin, Heilongjiang, Anhui, Jiangxi, Henan, Hubei, and Hunan; Eastern: Beijing, Tianjin, Hebei, Liaoning, Shanghai, Jiangsu, Zhejiang, Fujian, Shandong, Guangdong, and Hainan. **(A)** COPD and **(B)** Asthma.

The analysis for COPD and asthma, two major contributions to CRD, showed substantial provincial variations between male and female patients, urban and rural residency, occupation, education, and marital status in 2020 (Figure 4). The factors associated with POD caused by COPD and asthma were similar to the general trends of CRD. For COPD-related deaths, individuals who lived in an urban setting, were unmarried, have a higher education level, and were from regions with higher GDP and GINI index were more likely to die in hospitals than their counterparts. For asthma-related deaths, the female gender and receiving college and above education were significantly associated with a higher likelihood of dying in hospitals.

## Discussion

Using data from the NMSS in China, this study revealed the distribution and the trends of POD among Chinese decedents who reported CRD as the underlying cause of death between 2014 and 2020. Our retrospective data analysis showed that home was the major POD among CRD deaths in China, followed by medical and healthcare institutions and on the way to hospitals. Such a pattern was not changed during 2014–2020. We also observed disparities in POD according to both individual demographic and socioeconomic characteristics, and contextual factors in socioeconomic development. These findings demonstrated the complex factors associated with POD in patients with CRD, and shed light for policymakers and practitioners on the need for EOL care.

To our knowledge, this is the first study to demonstrate the pattern and trends of POD among CRD decedents in China. Our study demonstrated that home was the most common POD of CRD deaths (more than 80%) in China during 2014–2020, which was extremely higher than in many developed countries (9). On the one hand, culture is one key reason for such discrepancy. Chinese have a traditional cultural preference for dying at home, which is called “falling leaves to the roots,” resulting in a high proportion of home death (24, 25). On the other hand, the high and stable trends of POD among CRD deaths may suggest the potential insufficient accessibility and affordability of healthcare in China. Previous research from other Asian countries including South Korea (26) and Japan (27) observed greater institutionalization of dying and a decrease in home deaths as a result of rapid economic transformation, alterations in household structure, and growth in medical services particularly hospital-based care. In some of the developed countries, for instance, in the US, there was a trend of increasing home deaths or nursing home deaths due to the improvement of palliative care and the introduction of the Medicare payment system (28–30). Although POD cannot fully indicate the quality or experience of EOL care, our study indicated an existing gap and mismatch between health needs and healthcare services in China.

In addition, the proportion of home deaths among CRD deaths was higher than home deaths caused by other health conditions in China. For instance, previous analysis of cardiovascular disease deaths showed about 77% of home deaths during 2008–2020 in China (14). This finding was different from previous findings in the US, where CRD had the greatest odds of hospital deaths (30) as patients with CRD may experience acute deteriorations in symptoms and have higher needs for hospital-based services at the end of life (31). The disproportion of home deaths across diseases may reflect the lack of awareness of people with CRD and the limited accessibility and availability of acute-stage care for people with CRD in China.

As noted, disparities between individuals and across regions exist for POD among CRD-caused deaths in China. We identified some of the individual- and contextual-level factors that were associated with the POD of CRD deaths in China, which provide some explanations about the variation across provinces and populations. At the individual level, our study found that sex, age, marital status, and education level were key factors associated with home deaths. This finding was consistent with previous studies, which found that death at home was more likely for individuals who were female, older, and had a higher level of education among patients with lung diseases (12, 32). We also observed a higher likelihood of home deaths among rural residents and in regions with lower socioeconomic development status, including the lower level of GDP, number of beds in healthcare institutions, and average years of educational attainment. Similar factors were also identified in some of the previous studies, which also found that POD was associated with socioeconomic deprivation and urbanization level (33). The variations of POD found in our study could support our understanding of the characteristics of CRD deaths in China and illustrate the potential need for EOL care.

Our study has several strengths. We used data from the NMSS with the capability to offer both national and provincial representativeness. Key variables used in the study, including the cause of death and POD have been verified by following a unified procedure to ensure the quality of data. Moreover, we used multilevel analysis to demonstrate both individual-level and spatial variation of POD among CRD deaths. However, our study findings should also be interpreted with caution. Our study assessed demographic and regional contextual factors that may be associated with POD, but some other important factors, such as individual preferences of POD, awareness of CRD, availability, accessibility and utilization of care for CRD, treatment received before deaths, attendance of nursing staff, or other related factors, were not measured or collected in the NMSS, thus was not considered in the analysis. Future studies could further examine the impact of these factors on CRD deaths. Besides, there might be a risk of committing ecological fallacy for variables measured at the area level.

In conclusion, a high proportion of CRD deaths occurred at home and both individual and contextual factors were associated with POD among CRD deaths. Given the aggravation of the aging population and the increasing significant burden of CRD in China, there will be increasing needs from patients with end-stage CRD who need hospital care. To better allocate healthcare resources and promote social and health equity, policymakers and practitioners need to consider the influence of social determinants on POD. Future research should further understand the reasons for the high proportion of home deaths to promote accessibility and high-quality EOL care.

## Data availability statement

The datasets generated and/or analyzed during the current study are not publicly available due to data sharing regulations established by the Chinese Center for Disease Control and Prevention on National Mortality Surveillance System. Relevant report could be found at: https://ncncd.chinacdc.cn/xzzq_1/202101/t20210111_223706.htm. The original datasets are available from the corresponding authors on reasonable request.

## Author contributions

XT, MZ, and YL contributed to the conception and design of the study and interpretation of the results and drafted the manuscript. WW and PY contributed to the acquisition of the data. XT, WW, XZ, EG, and PY contributed to the revision of the manuscript for important intellectual content. WW performed the statistical analysis. All authors read, revised, and approved the final manuscript.
