# Supplementary material for: Place of death among individuals with chronic respiratory diseases in China: Trends and associated factors between 2014 and 2020
Source: Front Public Health. 2023 Feb 20;11:1043534. doi: 10.3389/fpubh.2023.1043534 (PMC9987852; doi:10.3389/fpubh.2023.1043534)
Supplement: Supplementary file 1 [file Data_Sheet_1.docx]

### Supplementary Table 1. Included number of NMSS surveillance points and CRD deaths nationwide, 2014-2020

| **Year** | **No. of total NMSS points** | **No. of included NMSS points** | **No. of included CRD deaths in NMSS** |
| --- | --- | --- | --- |
| 2014 | 605 | 491 | 179177 |
| 2015 | 605 | 484 | 160127 |
| 2016 | 605 | 499 | 165295 |
| 2017 | 605 | 509 | 161166 |
| 2018 | 605 | 512 | 158315 |
| 2019 | 605 | 520 | 153357 |
| 2020 | 605 | 522 | 134308 |

### Supplementary Table 2a. Total CRD

| **POD** | **2014** | **2015** | **2016** | **2017** | **2018** | **2019** | **2020** | **Annual change (%, 95%CI) ^a^** |
| --- | --- | --- | --- | --- | --- | --- | --- | --- |
| Total (N) | 178641 | 160120 | 165295 | 160342 | 158315 | 152874 | 134380 | **--** |
| Medical and healthcare institutions (%) | 14.01 | 15.25 | 14.99 | 14.72 | 15.21 | 15.43 | 15.16 | 0.98 (-0.02, 1.98) |
| Home (%) | 83.53 | 82.58 | 82.84 | 83.23 | 82.55 | 82.40 | 82.61 | -0.15 (-0.30, 0.01) |
| Nursing homes (%) | 1.00 | 0.77 | 0.67 | 0.65 | 0.65 | 0.65 | 0.61 | -6.61 (-10.35, -2.88) |
| On the way to hospitals (%) | 0.66 | 0.80 | 0.91 | 0.81 | 1.04 | 1.05 | 1.12 | 8.09 (5.18, 10.99) |
| Others/Unknown (%) | 0.81 | 0.59 | 0.59 | 0.58 | 0.55 | 0.47 | 0.50 | -7.04 (-10.44, -3.65) |
| ^a^ The Poisson models were adjusted for age at death, sex and study area (province). | | | | | | | | |

### Supplementary Table 2b. COPD

| **POD** | **2014** | **2015** | **2016** | **2017** | **2018** | **2019** | **2020** | **Annual change (%, 95%CI) ^a^** |
| --- | --- | --- | --- | --- | --- | --- | --- | --- |
| Total (N) | 170967 | 153229 | 157974 | 153008 | 150587 | 144765 | 126924 | -- |
| Medical and healthcare institutions (%) | 13.51 | 14.77 | 14.49 | 14.21 | 14.64 | 14.73 | 14.42 | 0.72 (-0.37, 1.80) |
| Home (%) | 84.12 | 83.13 | 83.41 | 83.81 | 83.18 | 83.17 | 83.42 | -0.10 (-0.26, 0.06) |
| Nursing homes (%) | 0.94 | 0.74 | 0.64 | 0.62 | 0.62 | 0.60 | 0.58 | -6.78 (-10.21, -3.36) |
| On the way to hospitals (%) | 0.65 | 0.80 | 0.90 | 0.81 | 1.03 | 1.06 | 1.11 | 8.23 (5.35, 11.10) |
| Others/Unknown (%) | 0.78 | 0.56 | 0.55 | 0.55 | 0.53 | 0.43 | 0.47 | -7.45 (-11.26, -3.63) |
| ^a^ The Poisson models were adjusted for age at death, sex and study area (province). | | | | | | | | |

### Supplementary Table 2c. Asthma

| **POD** | **2014** | **2015** | **2016** | **2017** | **2018** | **2019** | **2020** | **Annual change (%, 95%CI) ^a^** |
| --- | --- | --- | --- | --- | --- | --- | --- | --- |
| Total (N) | 4772 | 4213 | 4278 | 4279 | 4323 | 4341 | 3824 | -- |
| Medical and healthcare institutions | 13.66 | 14.34 | 13.44 | 13.39 | 13.09 | 14.01 | 14.17 | 0.13 (-1.22, 1.48) |
| Home | 81.45 | 81.49 | 82.16 | 82.75 | 82.74 | 81.69 | 81.62 | 0.07 (-0.21, 0.34) |
| Nursing homes | 2.35 | 1.61 | 1.57 | 1.36 | 1.32 | 1.66 | 1.39 | -6.03(-12.00, -0.06) |
| On the way to hospitals | 1.05 | 1.26 | 1.26 | 1.17 | 1.76 | 1.34 | 1.60 | 6.15 (1.31, 10.98) |
| Others/Unknown | 1.49 | 1.31 | 1.57 | 1.33 | 1.09 | 1.31 | 1.23 | -3.36 (-7.24, 0.52) |
| ^a^ The Poisson models were adjusted for age at death, sex and study area (province). | | | | | | | | |
